# Nodular Pulmonary Amyloidosis Associated With Sjögren's Syndrome

**DOI:** 10.7759/cureus.105145

**Published:** 2026-03-13

**Authors:** Raquel Borrego, André Soveral, Sandra André

**Affiliations:** 1 Pulmonology, Unidade Local de Saúde de Lisboa Ocidental, Lisbon, PRT; 2 Pulmonology, Unidade Local de Saude de Lisboa Ocidental, Lisbon, PRT

**Keywords:** amyloidosis, autoimmune disease, incidental lung nodule, pulmonary nodular amyloidosis, sjögren syndrome

## Abstract

Nodular pulmonary amyloidosis is a rare entity characterized by extracellular deposits of amyloid protein in the pulmonary parenchyma, usually with a benign prognosis and often asymptomatic. We report a case of a 61-year-old man with a prior diagnosis of Sjögren’s syndrome who, during routine follow-up, was found to have multiple asymptomatic pulmonary nodules in the right lung, with uptake on positron emission tomography-computed tomography (PET CT) (SUVmax 2.11). Surgical resection was performed, and histological analysis revealed nodular deposits of amyloid protein with kappa light chain predominance, without evidence of systemic involvement. This case highlights the importance of considering nodular pulmonary amyloidosis in the differential diagnosis of pulmonary nodules in patients with Sjögren’s syndrome, emphasizing the need for histological evaluation to avoid misdiagnosis and inappropriate treatment.

## Introduction

Pulmonary amyloidosis is a rare disorder characterized by extracellular deposition of amyloid protein in the lungs, which can be localized or part of systemic amyloidosis. In the lung, this entity can present as nodular, diffuse alveoloseptal, or tracheobronchial involvement. Nodular pulmonary amyloidosis is defined by one or more localized nodular deposits of amyloid protein in the lungs [1,2]. Nodular pulmonary amyloidosis often does not involve other organs and has a benign prognosis. In most cases, patients are asymptomatic, and nodules are incidentally detected on imaging studies [2,3]. However, this condition may be associated with lymphoproliferative disorders or autoimmune diseases such as Sjögren’s syndrome [2,4], as in this clinical case. Moreover, it may mimic other diagnoses, including malignancy [5]. Therefore, histological diagnosis and investigation of associated conditions are essential.

## Case presentation

**Figure 1:**
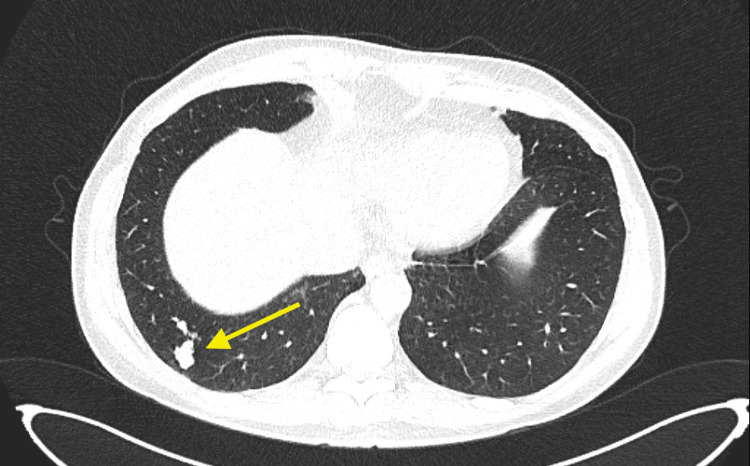
Chest CT with multiple nodules Chest CT with multiple nodules 0.8-2.0 cm. Arrow points to a solid nodule in the right lung.

A 61-year-old man, non-smoker, with a longstanding (>10 years) diagnosis of Sjögren’s syndrome, positive for antinuclear antibodies (ANA), anti-Sjögren’s-syndrome-related antigen A antibodies (anti-SSA [Ro60]), and anti-Sjögren’s-syndrome-related antigen B antibodies (anti-SSB [La]), underwent routine computed tomography (CT) of the chest, which revealed multiple solid peripheral nodules with lower predominance in the right lung measuring 0.8-2.0 cm (Figure 1). 

**Figure 2:**
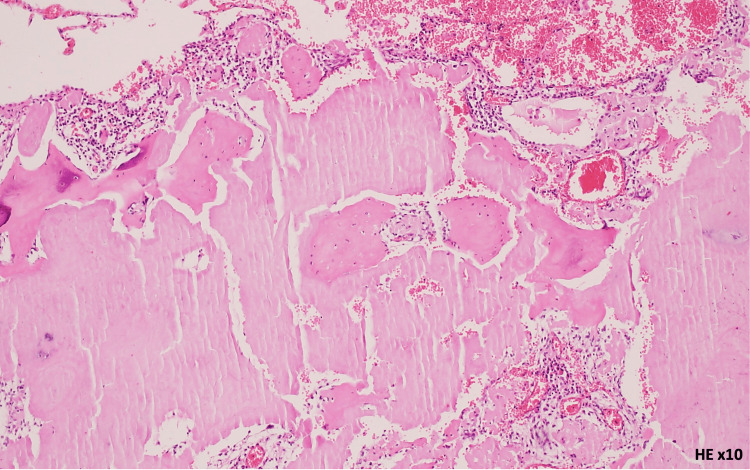
Amyloid substance: amorphous eosinophilic material (hematoxylin-eosin stain, ×10 magnification)

**Figure 3:**
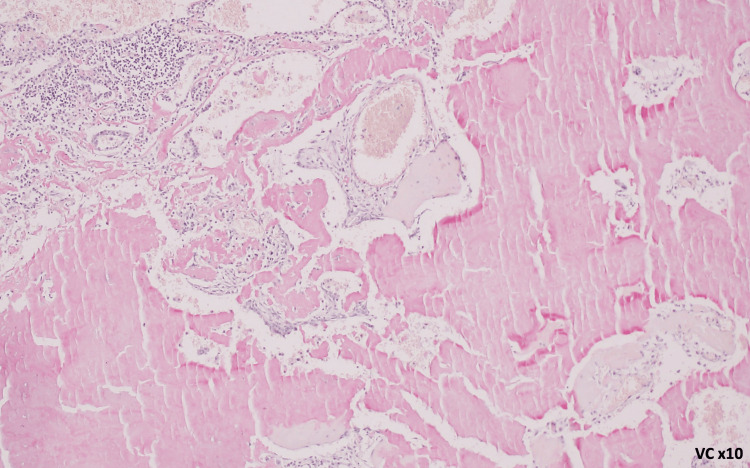
Amyloid substance: amorphous material highlighted in red by the Congo red histochemical technique (×10 magnification)

**Figure 4:**
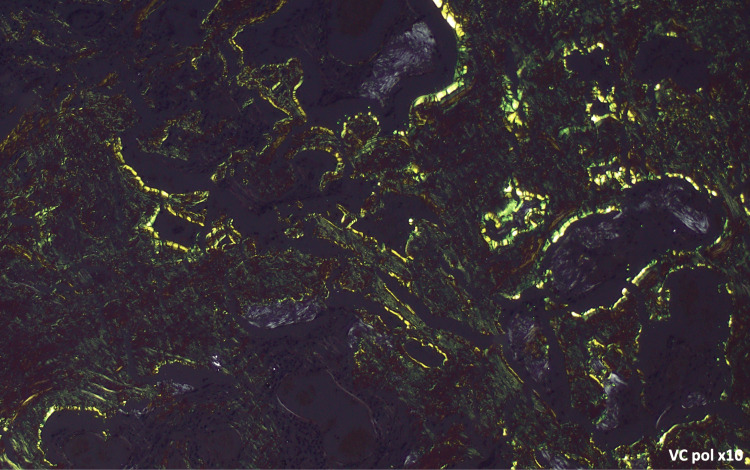
Amyloid substance: amorphous material demonstrated by Congo red histochemical staining with apple-green birefringence under polarized light (×10 magnification)

The patient had no respiratory symptoms, sicca symptoms, or abnormal findings on physical examination, and no family history of pulmonary disease. The patient was not under any medication. Given the presence of these nodules and the suspicion of malignancy, PET/CT was performed, showing mild homogeneous uptake in the same nodules (SUVmax 2.11). A transthoracic lung biopsy was considered but was technically not feasible due to the interposition of anatomical structures. Following multidisciplinary discussion, the patient underwent atypical resection of the right upper and lower lobes with lymph node sampling via video-assisted thoracoscopic surgery (VATS) without immediate or later complications. Histopathological analysis of the lung parenchyma revealed nodules composed of eosinophilic, amorphous material, occasionally associated with calcifications. Amyloid deposits were confirmed by Congo red staining, demonstrating apple-green birefringence under polarized light, with a predominance of kappa chains on immunohistochemistry, without features suggestive of mucosa-associated lymphoid tissue lymphoproliferation or plasmacytoma (Figures 2-4). The biopsy is consistent with nodular pulmonary amyloidosis.

Systemic evaluation followed with blood analysis, including a liver panel, bone marrow evaluation, serum immunoglobulins, and serum protein electrophoresis (Tables 1-3). The patient also underwent an echocardiogram, which was normal. Since surgery, the patient has remained under follow-up (1.5 years) without development of new nodules.

**Table 1:** Blood and urine evaluation This table provides an organized summary of the patient’s laboratory results, categorized into five major clinical areas: hematology, kidney and liver function, inflammatory markers, immunology, and autoimmune screening.

Test items	Laboratory results	Reference range
White blood cell (×10^9^/L)	5.6	4.0-10.0
Hemoglobin (g/L)	124	130-170
Platelet (×10^9^/L)	208	150-400
Urea (mg/dL)	46	17-49
Creatinine (mg/dL)	1.2	0.7-1.3
Sodium (mEq/L)	140	136-145
Potassium (mEq/L)	4.14	3.5-5.1
Calcium (mg/dL)	10.1	8.4-10.2
Total bilirubin (mg/dL)	0.23	<1.40
Aspartate transaminase (UI/L)	24	<40
Alanine transaminase (UI/L)	28	<41
Gamma-glutamyl transferase (UI/L)	11	10-71
Alkaline phosphatase (UI/L)	51	40-130
C-reactive protein (mg/dL)	<0.10	<0.50
Erythrocyte sedimentation rate (mm/h)	36	<15
Immunoglobulin G (mg/dL)	2180	600-1500
Immunoglobulin A (mg/dL)	347	50-400
Immunoglobulin M (mg/dL)	57.9	50-300
Serum protein electrophoresis	Polyclonal hypergammaglobulinemia	Normal pattern with no monoclonal bands
Serum κ light chain (mg/dL)	524	170-370
Serum λ light chain (mg/dL)	220	90-210
κ/λ ratio	2.38	1.35-2.65
Serum κ free light chain (mg/L)	39	6.7-22.4
Serum λ free light chain (mg/L)	37.6	8.3-27
Fκ/Fλ ratio	1.04	0.31-1.56
Rheumatoid factor (IU/mL)	51	<15
Anti-nuclear antibody	>1/1280	Negative
Anti-SS-A/Ro60 antibody	Strong positive	Negative
Anti-SS-A/Ro52 antibody	Strong positive	Negative
Anti-SS-B/La antibody	Strong positive	Negative
NT-pro Brain natriuretic peptide (pg/mL)	45	<300
Summary of urine analysis	Negative for glucose, proteins, ketone bodies, and hemoglobin	Negative

**Table 2:** Myelogram This table provides the results of myelogram that was used to help exclude myeloproliferative neoplasms.

Cells	Laboratory results	Reference range
Blast	2.2%	0.5-2.0%
Promyelocyte	2.2%	2.0-4.6%
Myelocyte	6.4%	8.1-16.9%
Metamyelocyte	7.3%	7.1-25.3%
Neutrophil	24.8%	16.5-36.8%
Eosinophil	2.9%	1.2-6.2%
Basophil	0.2%	0.0-0.2%
Monocyte	4.4%	0.2-5.0%
Lymphocyte	13.7%	10.8-22.7%
Plasma cell	3.7%	0.2-3.9%
Erythroblast	32.2%	20-50%
Myeloid/erythroid ratio	1.6	1.2-5.0
Myeloid series	No maturation/morphologic abnormalities	
Lymphoid series	Small lymphocytes with mature morphology	
Erythroid series	No maturation/morphologic abnormalities	
Megakaryocyte series	Normal number and without dysmorphias	

**Table 3:** Bone marrow evaluation Additional hematologic evaluation. Additional studies of bone marrow (immunophetyping and histochemical) to exclude infiltration by myeloproliferative disease and amyloid protein.

Cells	Laboratory results	Reference range
Bone marrow immunophenotyping		
Granulocytes	64.5%	50-65%
Lymphocytes	13.9%	10-20%
B lymphocytes (CD19)	3.55%	3-10%
Slg κ	0.56%	0.40-0.60%
Slg λ	0.36%	0.20-0.40%
κ/λ ratio	1.56	0.8-3
Plasma cells	0.38%	<0.40%
Comment	No abnormal population of lymphocytes found	
Bone marrow biopsy		
Amyloid protein (red Congo stain)	Negative	Negative

## Discussion

Sjögren's syndrome is a chronic systemic autoimmune exocrinopathy characterized by lymphocytic infiltration and progressive destruction of exocrine glands, predominantly the lacrimal and salivary glands. Pathologically, it is defined by focal lymphocytic sialadenitis with periductal aggregates of CD4⁺ T cells, B cells, and plasma cells within salivary gland tissue, often accompanied by B-cell hyperactivity and autoantibody production, particularly anti-SSA/Ro and anti-SSB/La antibodies.

The treatment for Sjögren syndrome is individualized and depends on the severity and type of manifestations. The first line of treatment is symptomatic (sicca symptoms and pain), and in severe extraglandular manifestations, systemic immunosuppressive therapy is used. The most frequent complication of systemic immunosuppressive therapy is infection, followed by malignancy. Also, patients with primary Sjögren's syndrome have a two-to-four-fold increased risk of overall malignancy compared with the general population and a 10- to 20-fold increased risk of lymphoma.

As a systemic disease, Sjögren's syndrome is associated with multiple pulmonary manifestations, with interstitial lung disease being the most frequent [6]. Nodular pulmonary amyloidosis as a manifestation of Sjögren's syndrome is rarely reported. Many cases of pulmonary amyloidosis, especially asymptomatic ones, are incidentally detected on imaging studies [7].

It is essential to differentiate nodular pulmonary amyloidosis from other causes of nodules, including malignancy and infection, given the implications for treatment and prognosis [8].

On imaging, nodular pulmonary amyloidosis may present as solitary or multiple nodules, sometimes with cavitation or calcifications, generally showing slow growth. However, these findings can mimic primary or metastatic neoplasms as well as infections, obscuring the differential diagnosis. Therefore, imaging alone is insufficient for diagnosis, as different entities can produce similar findings on CT and PET scans [9]. Definitive diagnosis of nodular pulmonary amyloidosis requires histopathological confirmation of amyloid deposition using Congo red staining, which demonstrates apple-green birefringence under polarized light [1-3]. In addition to diagnostic confirmation, it is crucial to exclude systemic involvement.

In the present clinical case, the development of pulmonary nodules in an asymptomatic patient with an autoimmune disease raised concern for malignancy. Also, due to the number and size of the nodules, the suspicion of malignancy was elevated.

For the study of malignancy, the patient underwent PET/CT, which showed mild homogeneous uptake in the same nodules (SUVmax 2.11). There is no universal cutoff value for the maximum standardized uptake value (SUVmax) in PET scans, as the optimal threshold varies by clinical context, organ system, and disease. However, in the evaluation of mediastinal and hilar lymph nodes for malignancy, a commonly used cutoff is 2.5, but this value is associated with high sensitivity and low specificity.

After ascertaining the need for nodule sampling, a multidisciplinary discussion between Radiology, Pulmonology, and Thoracic Surgery followed. Due to the location of the nodules, percutaneous thoracic biopsy was not feasible because of the interposition of other anatomical structures, and bronchial fibroscopy could not reach the nodules. Therefore, it was determined that a surgical approach was the best option to sample the nodules.

The patient underwent atypical resection of the right upper and lower lobes with lymph node sampling via VATS, which obtained the needed material.

The malignant hypothesis was excluded by histological evaluation of the surgical specimen. The histology revealed deposits of amyloid protein, raising the question of pulmonary versus systemic amyloidosis (Figures 2-4).

Amyloidosis is a group of disorders characterized by extracellular deposition of misfolded proteins (amyloid fibrils) in tissues and organs, leading to progressive disruption of normal structure and function. The major biochemical types are classified by their deposits: AL (light-chain) amyloidosis, ATTR (transthyretin), AA (amyloid A protein), Aβ₂M (dialysis-related), and Aβ in Alzheimer's disease. Clinically, amyloidosis can be categorized by distribution as systemic or localized.

To exclude systemic amyloidosis, the patient underwent echocardiography and NT-proBNP assay to exclude cardiac involvement, a liver panel with normal hepatic function to exclude liver involvement, and urine and renal function testing to exclude kidney involvement. Hematologic evaluation was also performed with immunologic and serologic blood work plus bone marrow study.

In the present case, the patient had no involvement of other organs, so pulmonary amyloidosis was assumed. Most cases of nodular pulmonary amyloidosis associated with Sjögren's syndrome are localized, and treatment is generally conservative, as most lesions remain stable over time without progression. Surgical excision may be considered for diagnostic or symptomatic lesions and is usually curative. Long-term prognosis is excellent, with rare recurrence or progression to systemic disease [1,5].

Long-term follow-up with chest CT, complete blood count, renal and liver function tests, and NT-proBNP is recommended, as rare cases progress or are associated with mucosa-associated lymphoid tissue lymphomas [10].

## Conclusions

Nodular pulmonary amyloidosis should be considered in the differential diagnosis of pulmonary involvement in Sjögren’s syndrome and in the evaluation of pulmonary nodules. This case illustrates a rare association between these two entities.

Even though the biopsy may be technically challenging, histology is required to avoid misdiagnosis and overtreatment.
